# Identification of CD4-Binding Site Dependent Plasma Neutralizing Antibodies in an HIV-1 Infected Indian Individual

**DOI:** 10.1371/journal.pone.0125575

**Published:** 2015-05-11

**Authors:** Lubina Khan, Muzamil Ashraf Makhdoomi, Sanjeev Kumar, Ambili Nair, Raiees Andrabi, Brenda E. Clark, Kate Auyeung, Jayanta Bhattacharya, Madhu Vajpayee, Naveet Wig, Ralph Pantophlet, Kalpana Luthra

**Affiliations:** 1 Department of Biochemistry, All India Institute of Medical Sciences, New Delhi, India; 2 Faculty of Health Sciences, Simon Fraser University, Burnaby, British Columbia, Canada; 3 HIV Vaccine Translational Research Laboratory, THSTI-IAVI HIV Vaccine Design Program, Translational Health Science and Technology Institute, Gurgaon, Haryana, India; 4 Department of Microbiology, All India Institute of Medical Sciences, New Delhi, India; 5 Department of Medicine, All India Institute of Medical Sciences, New Delhi, India; Emory University, UNITED STATES

## Abstract

Dissecting antibody specificities in the plasma of HIV-1 infected individuals that develop broadly neutralizing antibodies (bNAbs) is likely to provide useful information for refining target epitopes for vaccine design. Several studies have reported CD4-binding site (CD4bs) antibodies as neutralization determinants in the plasma of subtype B-infected individuals; however there is little information on the prevalence of CD4bs specificities in HIV-infected individuals in India. Here, we report on the presence of CD4bs antibodies and their contribution to virus neutralization in the plasma from a cohort of HIV-1 infected Indian individuals. Plasma from 11 of the 140 HIV-1 infected individuals (7.9%) studied here exhibited cross-neutralization activity against a panel of subtype B and C viruses. Analyses of these 11 plasma samples for the presence of CD4bs antibodies using two CD4bs-selective probes (antigenically resurfaced HXB2gp120 core protein RSC3 and hyperglycosylated JRFLgp120 mutant ΔN2mCHO) revealed that five (AIIMS 617, 619, 627, 642, 660) contained RSC3-reactive plasma antibodies and only one (AIIMS 660) contained ΔN2mCHO-reactive antibodies. Plasma antibody depletion and competition experiments confirmed that the neutralizing activity in the AIIMS 660 plasma was dependent on CD4bs antibodies. To the best of our knowledge, this is the first study to report specifically on the presence of CD4bs antibodies in the plasma of a cohort of HIV-1 infected Indian donors. The identification of CD4bs dependent neutralizing antibodies in an HIV-1 infected Indian donor is a salient finding of this study and is supportive of ongoing efforts to induce similar antibodies by immunization.

## Introduction

Development of an effective vaccine against human immunodeficiency virus (HIV-1) continues to be a global challenge. A major focus is on the design of envelope immunogens that can elicit a potent broadly neutralizing antibody (bNAb) response. It is well known that all the neutralizing antibody (NAb) response in HIV-1 infected individuals is directed against the envelope glycoprotein of HIV-1 [1–4]. Individuals infected with HIV mount varied immune responses and the plasma antibodies of 7–25% of the patients have been reported to efficiently neutralize different HIV-1 subtypes [5–9]. A number of detailed sera mapping studies have revealed epitopes on the viral envelope glycoprotein targeted by bNAb responses [5, 10–15].

Over the last two decades, and particularly in the last 5–7 years with the advent of high throughput techniques, a sizeable number of bNAbs with different specificities have been isolated from HIV-1 infected individuals. These include specificities to the CD4bs, exemplified by antibodies b12, 3BNC117 and the VRC01 family of antibodies [16–20], glycan dependent trimer specific epitopes spanning the V2V3 region, recognized by antibody PG9 and the CH01 and PGT145 antibody families [21–23], glycan dependent epitopes at the base of the V3 region, represented by antibody 2G12 and the PGT121 and PGT128 antibody families [24–27], quaternary glycan dependent epitopes that span the gp120/gp41 interface, exemplified by antibody 35O22 and the PGT151 family of antibodies [28–30], and the membrane-proximal external region of gp41, targeted by antibodies 2F5, 4E10 and 10E8 [31–36].

One of the most studied regions on the HIV-1 envelope is the CD4bs, because of its highly conserved nature as the site responsible for binding to the CD4 receptor on target cells [37–39]. Over the last several years, several CD4bs-specific antibodies have been described that can efficiently neutralize a broad range of HIV strains at high potency (reviewed in [40, 41]). The antibody VRC01, for example, neutralizes as much as 90% of HIV-1 strains and binds in a manner analogous to the CD4 receptor [17]. CD4bs antibodies have been reported in a considerable number of broadly cross neutralizing plasma (CNP) from HIV-1 infected South African individuals [10–13, 42]. A recent longitudinal study conducted in the CHAVI, CAPRISA and Amsterdam cohorts revealed that majority of the plasma samples contained CD4bs antibodies, suggesting high immunogenicity of the CD4bs region during the course of infection [42]. The somewhat high prevalence of CD4bs antibodies in HIV-1 infected individuals makes this region a prime target epitope for immunogen design. Further, screening of potential HIV-1 infected individuals from different populations for the presence of CD4bs antibodies, particularly if responsible for conferring cross-neutralizing activity, will furnish supportive information for the probability of inducing similar antibodies upon immunization.

India ranks third after South Africa and Nigeria in the HIV-1 epidemic with approximately 2.4 million infected individuals[43]. Limited information exists on the plasma antibody specificities of Indian HIV-1 infected individuals [44], and none of the studies from this region have addressed CD4bs antibodies as neutralizing determinants. While the majority of HIV-1 infections in the Indian population are attributed to the subtype C, it is worth noting that the circulating subtype C envelope sequences in these individuals have distinct epitope specificities in comparison to the subtype C and non-subtype C viruses from other populations [44, 45].

Here, we present the first study to map the CD4bs antibody specificities of the neutralizing plasma samples of HIV-1 infected Indian individuals. This was done using RSC3, an antigenically resurfaced and stabilized protein derived from the HXB2 gp120 core that was used previously for the isolation of VRC01 [18] and ΔN2mCHO, a protein derived from JRFL gp120 onto which extra glycans have been incorporated to occlude the epitopes of non-CD4bs antibodies while maintaining exposure of the CD4bs. This so-called hyperglycosylated gp120 mutant binds several CD4bs antibodies with high affinity ([46, 47] and unpublished data). In addition to these two probes, HXB2 gp120 and its mutant HXB2 gp120-D368R were used to confirm the presence of CD4bs plasma antibodies. [39]

## Materials and Methods

### HIV-1 positive plasma samples and Ethics statement

A total of 140 HIV-1 positive adults were included in this study, of which the plasma neutralization data of 80 HIV-1 infected individuals was reported earlier [44]. Sixty HIV-1 infected individuals were recruited freshly for this study from the Department of Medicine, All India Institute of Medical Sciences (AIIMS), New Delhi, India. In addition, two serum samples (VC10042 and VC10071) from a previously described Vanderbilt/CFAR cohort [15] were included. A written informed consent was taken from all the individuals and the study was ethically approved by the ethics committee of AIIMS. Blood samples were drawn in EDTA vacutainers and the plasma was separated and stored at -80°C until use. Prior to assaying, the plasma samples were heat inactivated at 56°C for 1 h.

### Monoclonal Antibodies

The anti-HIV-1 monoclonal antibodies used in this study (b12, VRC01, and 447-52D) were obtained from NIH-AIDS Research and Reference Reagent Program (NIH ARRRP). Antibody 1418, specific for the capsid of human parvovirus B19, was kindly provided by Susan Zolla Pazner. F(ab)'2 b12 was generated from full-length b12 by cleavage with pepsin as described elsewhere [48]. F(ab)'2 was separated from undigested IgG and Fc fragments by protein A purification. Purity was confirmed by SDS-PAGE.

### Expression and Purification of CD4bs-selective Proteins

Plasmids encoding for recombinant proteins that allow for the identification of CD4bs antibodies were obtained from different sources: The resurfaced stabilized gp120 core protein RSC3 (plasmids were kindly provided by John Mascola, National Institute of Allergy and Infectious Diseases, USA) designed to preserve the structure of neutralizing epitopes overlapping the CD4bs and its mutant RSC3Δ371I, wherein deletion of the isoleucine at 371 position on RSC3 reduces the binding affinity of several, though not all, neutralizing antibodies to the CD4bs [18, 42]; HXB2 gp120 and its D368R mutant (plasmids were kindly provided by Joe Sodroski, Dana Farber Cancer Institute, USA) in which the replacement of Asp368 by Arg eliminates the binding of most CD4bs antibodies [17, 39]. The respective proteins were expressed from the plasmids by transient transfection using polyfectamine (Qiagen) in 293T cell lines. Supernatants were harvested after 5 days, filtered using 0.45 μm filters (Millipore) and stored at 4°C prior to purification. In case of RSC3and its Δ371I mutant, Ni-NTA beads (Qiagen) chromatography was used to purify the harvested proteins [18] whereas for HXB2 gp120 and its D368R mutant, lentil lectin beads (GE Healthcare) were used. The eluted fractions were collected, dialysed extensively against PBS and concentrated (Amicon 10kDa, Millipore). Purity of the proteins was checked by SDS-PAGE and functionality was checked by binding ELISA using specific bNAbs (VRC01 and b12, 447-52D). Wild-type JRFL gp120 and the hyperglycosylated mutant ΔN2mCHO were expressed from stably-transfected CHO-K1 cells and purified on Ni-NTA columns (Qiagen) as described elsewhere [47].

### Enzyme-linked immunosorbent assay (ELISA)

Initial screening for the presence of CD4bs antibodies in the HIV-1 infected plasma samples was done by ELISA. The proteins (RSC3, RSC3Δ371I, HXB2 gp120 and its D368R mutant, and JRFL gp120 and its hyperglycosylated mutant ΔN2mCHO) were coated onto ELISA plates (Corning) at 2 μg/ml in bicarbonate buffer (pH 9.6) and incubated at 4°C overnight. Plates were washed three times with 0.2% PBS-Tween, followed by blocking with RPMI (Hy-clone) supplemented with 15% fetal calf serum (FCS) (Hyclone) and 2% BSA (Sigma-Aldrich) and incubation at 37°C for 1 h. The plates were then washed as before and serial dilutions (starting at 1/30) of each plasma sample subsequently added and the plates incubated for 1 h at 37°C. The plates were then washed as before, incubated for 1.5 h at 37°C with alkaline-phosphatase conjugated anti-human IgG Fc (Southern Biotech; 1/2000 dilution) at, washed again as before, and then developed using alkaline phosphatase substrate (Sigma-Aldrich) solubilized in 10% diethanolamine buffer. The reactions were stopped after 20 min by the addition of 6N NaOH and the optical density read at 405 nm. The reported Max50 value is the 50% maximal binding of plasma antibodies that showed saturation. The plasma samples were tested with each of the envelope proteins (RSC3 and its RSC3Δ371I mutant, HXB2gp120 and its D368R mutant, and JRFL gp120 and its hyperglycosylated mutant ΔN2mCHO) and Max50 values were calculated as described previously [44].

For competition ELISA using soluble CD4, plates were coated with 1 μg/ml of anti-C5-antibody D7324 (Aalto) and incubated overnight at 4C. Plates were then washed three times with 0.05% PBST and blocked as described above. Following three washes with PBST, 1 μg/mL of JRFL gp120 was added and incubated for 1 h at room temperature (RT). Fixed concentration of sCD4 (20 μg/ml) was mixed with serial dilutions of control mAbs (b12 or 447-52D) (starting at 1 μg/ml) or antibodies eluted from adsorbed ΔN2mCHO coated beads (ΔN2mCHO eluate), added to the plates and incubated again for 1 h at RT. After three washings, the binding of the mAbs or ΔN2mCHO eluate antibodies to JRFL gp120 was detected using an alkaline-phosphatase conjugated anti-human IgG Fc (Southern Biotech; 1/2000 dilution) and phosphatase substrate as described above.

### Protein-magnetic bead coupling

For depleting the CD4bs antibodies from the plasma samples, CD4bs probes (RSC3, ΔN2mCHO, and also a pair of HXB2gp120 and HXB2gp120 D368R) and BSA (negative control protein) was conjugated to tosylactivated magnet MyOne dynabeads (Invitrogen). Coupling was performed according to the manufacturer’s instructions. Briefly, 1 mg of protein was coupled to 50 mg of tosylactivated magnet MyOne dynabeads. Coating was performed at 37°C in 1.25 ml of coupling buffer (0.1 M sodium borate buffer, pH 9.5, with 1 M ammonium sulphate) with gentle rocking for 10 to 12 h. The protein coated dynabeads were separated with a magnet and resuspended with 1.5 ml of blocking buffer (PBS supplemented with 0.5% BSA and 0.05% Tween 20) for 10 to 12 h. Next, the protein-coupled dynabeads were washed three times with 1.5 ml of washing buffer (PBS supplemented with 0.1% BSA and 0.05% Tween 20), then stored in 0.5 ml of storage buffer (PBS with 0.1% BSA, 0.05% Tween 20, and 0.02% sodium azide) at 4°C.

The specificity of each conjugated protein was verified by flow cytometry using the bNAbs b12, VRC01 and 447-52D. Typically, 5 to 10 μl of the protein-conjugated beads were washed thrice with 200 μl of 1XPBS containing 2% FCS and then bNAbs (VRC01 for RSC3-conjugated beads, b12 for HXB2 gp120 and ΔN2mCHO conjugated beads and 447-52D for HXB2 gp120-D368R conjugated beads) added at 5μg/ml (in 200 μl of PBS) and incubated at room temperature for 1 h. The beads were again washed thrice and stained with APC-conjugated goat anti-human Fc secondary antibody (BD Biosciences) at RT for 1 h in 200 μl of PBS. After washing, the extent of antibody binding to the beads was assessed by flow cytometry (BD-FACS, CANTO), and the data analysed using Flow Jo software (Tree Star, Inc., San Carlos, CA).

### Plasma Antibody adsorption / depletion assay

Protein-conjugated beads were washed thrice with Dulbecco modified Eagle medium (DMEM) containing 10% FCS (DMEM-FCS) followed by blocking with DMEM-FCS at RT for 30 min. Plasma samples from HIV-1 infected individuals were diluted at 1:30 in 1 ml of DMEM-FCS and incubated with 150μl of protein-conjugated beads at RT for 30 min. Three similar rounds of bead adsorption were performed to deplete CD4bs specific antibodies in the diluted plasma samples. After adsorption, the beads were separated from the diluted plasma samples using a magnet. The adsorbed plasma samples were centrifuged three times at 1500*g* for 5 min to remove any remaining beads, filtered with a 0.45 μm filter and then used for ELISA and neutralization assays. BSA-coated beads were used as negative controls in the adsorption assays. The percentage depletion of total gp120 specific CD4bs plasma antibodies (using specific probes) was checked by ELISA binding to the corresponding wild type gp120. RSC3, ΔN2mCHO, D368R were used for depletion. The plasma antibodies, before and after depletion, were tested for binding to HXB2 gp120 for both RSC3 and D368R depleted plasma and JRFL gp120 for ΔN2mCHO depleted plasma. The depletion by each probe is represented as a percentage of total gp120 specific antibodies using the formula: % depletion = 100-[100× (OD405 after 3 rounds of adsorption /OD405 before adsorption)] for RSC3 and ΔN2mCHO. For the HXB2 gp120- D368R depleted plasma; the % depletion was calculated as [100× (OD405 after 3 rounds of adsorption /OD405 before adsorption)]. BSA coupled beads were used as control. Two healthy HIV-1 seronegative plasma samples (designated A1 and A2) were used as ELISA controls.

For recovery of the plasma antibodies bound to the beads, beads were washed thrice with PBS. The beads were then treated with 100mM glycine-HCl buffer (pH 2.7) for 30s, followed by micro centrifugation for 30s. The beads were settled down at the bottom of the tube using a magnet. The eluted antibody fraction in the supernatant was collected into a separate tube and the pH adjusted to 7 using 1 M Tris buffer (pH 9.0). This entire procedure was repeated thrice. The eluted antibodies were diluted in DMEM and concentrated using a 10-kDa Amicon concentrator (Millipore). The beads were finally treated with glycine buffer (pH 2.2) to elute any remnant antibodies that had not eluted at pH 2.7. The eluted antibody fractions were then pooled and quantified using the Bradford’s reagent by ELISA.

### Neutralization assays

Neutralization efficiency of plasma antibodies was measured as a reduction in luciferase gene expression after a single round of infection of TZM-bl cells (obtained from the NIH ARRRP) with envelope pseudotyped viruses [49]. For pseudovirus production, 293T cells (6x10^5^ cells/well) were seeded in a 6-well plate and cultured in a humidified CO_2_ incubator overnight. The next day, the cells were co-transfected with 4 μg of a HIV-1 *env* expression plasmid and 8 μg of an *env*-deficient HIV-1 backbone vector (pSG3 ΔEnv), using polyfectamine (Qiagen) and then placed back in the humidified incubator. Pseudovirus containing culture supernatants were harvested 48h after transfection and filtered (0.45 μm) and stored at -80°C in 500 μl aliquots. TCID50 of aliquots containing pseudovirus was calculated and 200 TCID50 of virus was incubated with serially diluted heat-inactivated plasma samples (starting at 1/60) for 1h at 37C. Freshly trypsinized TZM-bl cells (in growth medium containing 25 μg/ml DEAE Dextran and Indinavir (1 mM) in case of primary isolates) at 10^5^ cells/well were added and plates incubated at 37C. Virus controls and cell controls were included. MuLV was used as a negative control. After 48 h, luciferase activity was measured using the Bright-Glow Luciferase Assay System (Promega Inc.). ID50 values of the plasma samples were calculated by determination of the plasma dilution that neutralized 50% of the infectious virus. Values were derived through a dose-response curve fit with non- linear function using the GraphPad prism software (San Diego, CA).

### Statistical Analyses

Statistical analyses were performed using Graph Pad Prism 5. Non-linear regression curve straight line was plotted using the method of least squares to determine the Max50 and ID50 values. Median reciprocal Max50 binding titres were compared using paired t test. P values < 0.05 were considered significant.

## Results

### Patient cohorts

This study was conducted with plasma from a total of 140 antiretroviral naïve HIV-1 infected Indian adults; 80 of these infected donors were profiled previously for viral neutralization activity [44]. Sixty chronically infected HIV-1 individuals (date of diagnosis ranging from 2 to 10 years) were recruited freshly for this study, comprising 28 males and 32 females within the age range of 18 to 50 years. All the 60 antiretroviral drug naïve individuals had a CD4 count ≥350 cells/mm^3^ with a median CD4 count of 436 (range 360–780) and tested negative for tuberculosis.

### Cross-neutralizing activity observed in a fraction of plasma antibodies tested

Neutralization activity in the plasma of the 60 HIV-1 seropositive individuals additionally recruited for this study was assessed against a panel of subtype B and subtype C pseudoviruses. Half of the plasma samples were tested against a panel of 22 pseudoviruses (17 subtype C and 5 subtype B; Table 1), the other half were checked against a panel of 13 pseudoviruses (6 subtype C, 6 subtype B and 1 subtype A) (Table 2). Of the 60 plasma samples, 8 (AIIMS 615, 616, 617, 619, 621, 627, 642 and 660), were able to neutralize >80% of all viruses tested (Tables 1 & 2). Of all the viruses tested, Du156.12 (tier 2; subtype C) was found to be the most susceptible to neutralization, as it was neutralized by all CNP samples at significantly high ID50 titres. In contrast, 3 samples (AIIMS 637, 639 and 643) were unable to neutralize even a single virus out of the 13 viruses tested (S1 Table). On the basis of the above results, the 8 CNP samples identified here (AIIMS 615, 616, 617, 619, 621, 627, 642 and 660; clinical data in Table 3) and 3 CNP samples (AIIMS 206, 239 and 249) from the cohort of 80 patients tested earlier [44], were analysed further for their CD4bs antibody specificities.

**Table 1:** Cross-neutralization activity of six select plasma samples from HIV-1 infected Indian individuals against a panel of subtype B and subtype C pseudoviruses.

Pseudovirus	Subtype	Tier	AIIMS-615	AIIMS-616	AIIMS-617	AIIMS-619	AIIMS-621	AIIMS-627
**JRFL**	**B**	2	*111*	*102*	*404*	*317*	**1233**	*145*
**JRCSF**	**B**	2	*174*	*178*	*637*	*618*	*245*	*768*
**PVO.4**	**B**	3	*114*	*112*	*500*	*73*	*246*	*142*
**SC422661.8**	**B**	2	*200*	*116*	*865*	*449*	*365*	*777*
**RHPA4259.7**	**B**	2	*756*	**1001**	**2939**	**2040**	**2738**	**3747**
**836**	**C**	1	**1225**	**1079**	**1054**	*295*	*584*	*609*
**25711**	**C**	1	*286*	*163*	*547*	*496*	*190*	*384*
**26191**	**C**	2	*370*	*330*	*456*	*374*	*267*	*848*
**16055**	**C**	2	*761*	*580*	**1387**	**1146**	**1027**	*400*
**16936**	**C**	2	*439*	*512*	*972*	*420*	**1932**	*570*
**4-2.J41**	**C**	2	*813*	*951*	**2264**	**1438**	**2092**	**1414**
**5-4.J16**	**C**	2	*177*	*114*	*350*	*302*	*474*	*153*
**7-J.20**	**C**	2	**3757**	**2732**	**1231**	**1573**	**1060**	*285*
**11-5.J12**	**C**	2	*561*	*401*	**1184**	*482*	**3395**	**2532**
**Du151.2**	**C**	2	*233*	*267*	*647*	**1249**	**1358**	*731*
**Du156.12**	**C**	2	**14580**	**5402**	**14183**	**3220**	**7133**	**8925**
**Du172.17**	**C**	2	*308*	*251*	*407*	*352*	*239*	*306*
**Du422.1**	**C**	2	*229*	*275*	*588*	*211*	*259*	*317*
**CAP45.G3**	**C**	2	*520*	*735*	*229*	*175*	*54*	**3243**
**CAP84.32**	**C**	2	*865*	*422*	**1043**	*400*	*729*	*145*
**CAP88.B5**	**C**	2	*347*	*340*	*145*	*378*	*154*	**1095**
**CAP239.G3**	**C**	2	*151*	*141*	**1131**	*321*	**1313**	<60

The neutralization breadth of plasma antibodies from 6 plasma samples obtained from HIV-1 infected ART-naive individuals from India were assessed against a panel of 22 subtype B and C viruses. The AIIMS ID of the plasma samples is provided at the top of the table. Plasma neutralization is shown as the reciprocal value of the ID50, which is the plasma dilution at which virus infectivity is inhibited to 50%. ID50>1000 (Bold), ID50 = 61–1000 (Italic) and 60, where ID50 was not reached. Each experiment was performed at least twice, independently.

**Table 2:** Cross-neutralization activity of two plasma samples from HIV-1 infected Indian individuals against a panel of 13 pseudoviruses (1 subtype A, 6 subtypes B and 6 subtype C viruses).

Pseudovirus	Subtype	Tier	AIIMS-642	AIIMS-660
**Q168ENVa2**	**A**	2	*210*	**1026**
**BaL.01**	**B**	1	**6210**	**1256**
**SF162**	**B**	1	*565*	*844*
**RHPA4259.7**	**B**	2	*356*	*123*
**TRO.11**	**B**	2	*234*	*205*
**SC422661.8**	**B**	2	*654*	*148*
**JRFL**	**B**	2	*250*	*540*
**ZM109F.PB4**	**C**	1	*85*	*80*
**25710**	**C**	1	*548*	*75*
**MW965**	**C**	1	**4358**	**5425**
**001428-2.42**	**C**	2	**5500**	**4236**
**Du156.12**	**C**	2	*460*	*367*
**Du422.1**	**C**	2	120	567

The neutralization breadth of plasma antibodies from 2 samples of HIV-1 infected ART-naive individuals from India were assessed against a panel of 13 subtype B and C viruses. Plasma neutralization is shown as the reciprocal value of the ID50, which is the plasma dilution at which virus infectivity is inhibited to 50%. Data presentation is as in Table 1. Each experiment was performed at least twice, independently.

**Table 3:** Clinical profile of 8 CNP of HIV-1 infected antiretroviral naive individuals.

Patient ID	Time since diagnosis	Gender	Age (Years)	CD4 count (cells/μl)	Viral Load (RNA copies/ml)
**AIIMS 615**	39 months	Male	34	564	45600
**AIIMS 616**	68 months	Male	26	391	101000
**AIIMS 617**	85 months	Male	42	504	1240
**AIIMS 619**	129 months	Female	30	655	3310
**AIIMS 621**	31 months	Female	32	714	5680
**AIIMS 627**	68 months	Male	34	360	20800
**AIIMS 642**	56 months	Male	35	465	25400
**AIIMS 660**	39 months	Female	31	580	5080

### Select CNP samples contain binding antibodies specific for the CD4bs, as revealed by selective proteins

**Fig 1:**
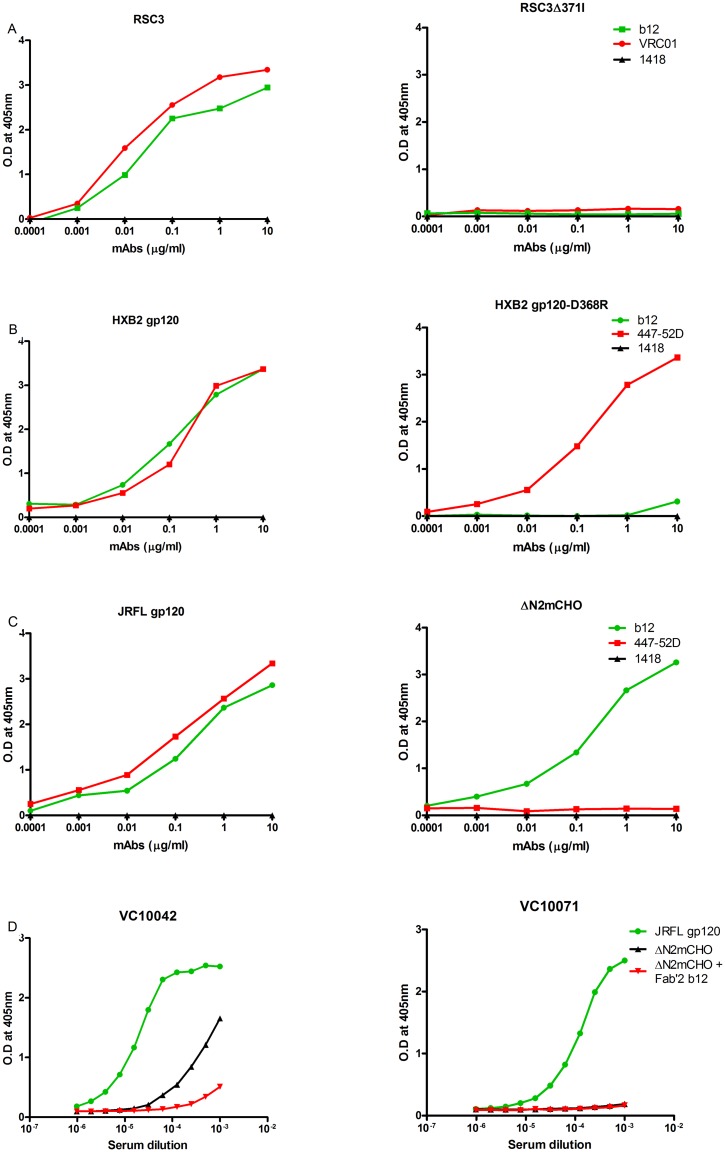
ELISA binding of CD4bs-selective probes with known bNAbs. To assess functionality of the CD4bs probes, binding of the recombinant proteins was checked with different concentrations of known bNAbs. Binding of the RSC3 and its RSC3Δ371I mutant was assessed with VRC01 and b12 (A), of HXB2 gp120 and its D368R mutant with b12 and 447-52D (B), JRFLgp120 and its hyperglycosylated mutant ΔN2mCHO with b12 and 447-52D (C). The selectivity of mutant ΔN2mCHO was confirmed using two characterized plasma samples (D). Human mAb 1418 to parvovirus B19 was used as negative control in all assays.

The selectivity of the CD4bs probes (RSC3 and RSC3 Δ371I, JRFL gp120 and ΔN2mCHO, HXB2 gp120 and HXB2 gp120-D368R) was first verified by ELISA with known bNAbs (Fig 1). As expected, both b12 and VRC01 bound the RSC3 probe but not RSC3Δ371I (Fig 1A). HXB2 gp120 exhibited binding with b12 and 447-52D, whereas its D368R mutant showed, as expected, binding with 447-52D but not with b12 (Fig 1B). JRFL gp120 was bound also by both 447-52D and b12, whereas hyperglycosylated mutant ΔN2mCHO only bound b12 (Fig 1C). The selectivity of mutant ΔN2mCHO was confirmed using two previously characterized plasma samples [15]; one, VC10042, is known to contain neutralizing antibodies to the CD4bs whereas the other, VC10071, only neutralizes SF162, presumably via non-CD4bs antibodies. As expected, both sera bound fairly strongly to JRFLgp120wt (Fig 1D). However, only serum VC10042 also bound mutant ΔN2mCHO. This binding was largely due to the CD4bs, as evidenced by near complete inhibition of the binding activity of VC10042 to ΔN2mCHO in the presence of F(ab)'2 b12, suggesting that ΔN2mCHO selectively presents just the CD4bs.

**Fig 2:**
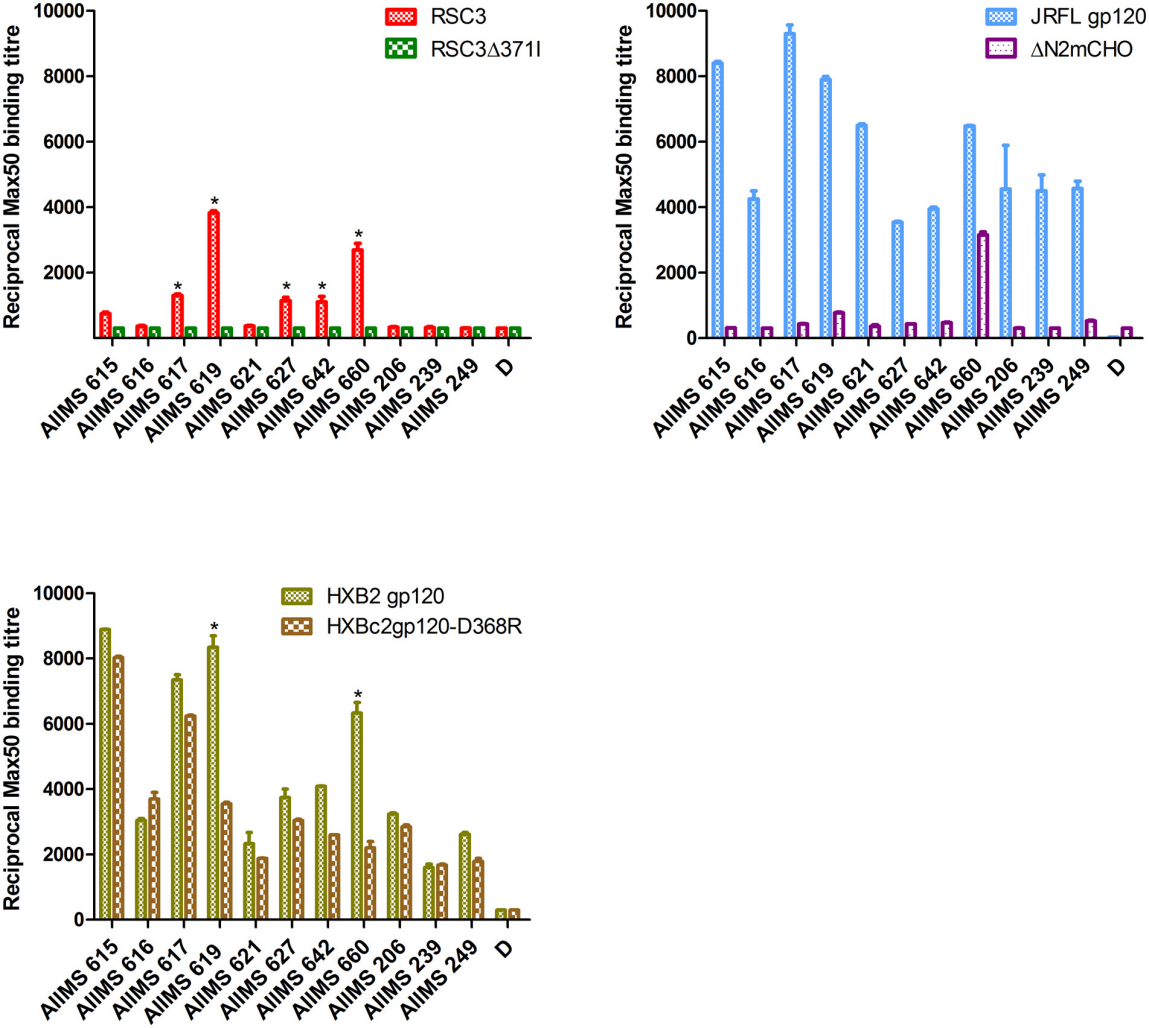
ELISA binding assay to determine the presence of CD4bs antibodies in the CNP samples. Eleven (AIIMS 615, 616, 617, 619, 621, 642, 660, 206, 239, 249) CNP samples were tested for the presence of CD4bs antibodies using three sets of probes. HXB2 gp120 and its D368R mutant, RSC3 and CD4bs-defective mutant RSC3Δ371I, JRFLgp120 and hyperglycosylated ΔN2mCHO, which were used at 2μg/ml. Reciprocal Max50 binding titres were calculated using the least square regression method. Plasma of one healthy seronegative individual (D) was used as assay control. * denotes P values <0.05.

Having verified the selectivity of the probes, we screened the 11 identified CNP samples for the presence of CD4bs antibodies (Fig 2). A plasma sample was considered to be reactive if its reciprocal Max50 binding titre was greater than 1000 for ΔN2mCHO and RSC3 and there was at least a 2-fold reduction with the respective CD4bs mutant for HXB2 gp120 and HXB2 gp120-D368R. Based on these criteria, 5 of the 11 plasma samples (AIIMS 617, 619, 627, 642, 660) bound RSC3, but did not bind the mutant RSC3Δ371I (P<0.05). AIIMS 660 and AIIMS 619 also demonstrated significant reduction in binding to the D368R mutant as compared to wild type HXB2 gp120 (P<0.05), further substantiating the presence of CD4bs antibodies in these samples. Strikingly, only plasma AIIMS 660 showed binding to the ΔN2mCHO hyperglycosylated mutant; AIIMS 619 showed little binding. Thus, of the 11 CNPs, 5(45%) contained RSC3-reactive CD4bs antibodies and, of these, only 1(9%) contained CD4bs antibodies able to recognize the highly selective mutant ΔN2mCHO. AIIMS 660 plasma antibodies exhibited a 9-fold reduction in binding affinity to mutant RSC3Δ371I compared to RSC3 and roughly a 3-fold reduction in binding to HXB2 gp120-D368R as compared to HXB2 gp120. The AIIMS 619 plasma antibodies bound 12-fold better to RSC3 as compared to the mutant RSC3Δ371I and roughly 2-fold worse to HXB2 gp120-D368R as compared to HXB2 gp120. Based on these results, AIIMS 660 and AIIMS 619 plasma samples were selected for further analyses.

To verify the presence of CD4bs antibodies in CNP samples AIIMS 660 and 619, depletion assays were performed using beads coated with the CD4bs-specific probes. For this, the HXB2 gp120 and its D368R mutant, RSC3 and ΔN2mCHO mutant were coupled to tosylactivated magnet MyOne dynabeads (Invitrogen). The integrity of the protein coupled beads was assessed by their binding reactivity to bNAbs by FACS analysis (S1 Fig).

**Fig 3:**
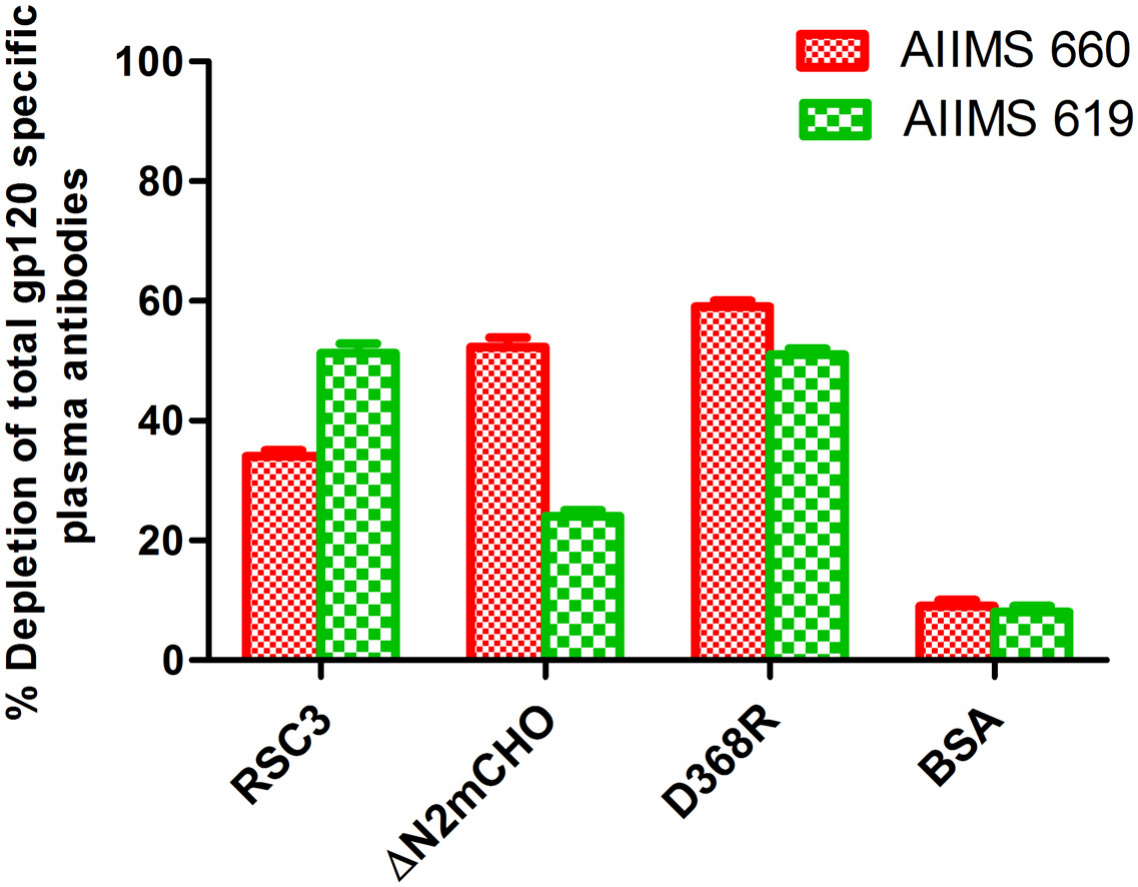
% depletion of gp120 specific antibodies from select CNP samples using CD4bs probes. The CNP samples that exhibited CD4bs specificities (AIIMS 660 and AIIMS 619) were depleted at 1:30 dilution in three rounds of incubation with the CD4bs probes (HXB2gp120, HXB2 gp120-D368R, RSC3, ΔN2mCHO and BSA) coupled magnetic beads. The depletion was verified by reduction in the ELISA binding of the depleted (flow-through) as compared to the undepleted plasma, with the corresponding wild type gp120. The plasma antibodies, before and after depletion, were tested for binding to the respective wild-type gp120 (HXB2 gp120 for both RSC3 and D368R depleted plasma and JRFL gp120 for ΔN2mCHO depleted plasma. Percent depletion of CD4bs plasma antibodies by each probe was calculated as: % depletion = 100-[100× (OD405 after 3 rounds of adsorption /OD405 before adsorption)] for RSC3 and ΔN2mCHO. For HXB2 gp120- D368R depleted plasma sample: % depletion = [100× (OD405 after 3 rounds of adsorption /OD405 before adsorption)]. BSA coupled beads were used as control.

The plasma samples AIIMS 660 and AIIMS 619 were then depleted using the aforementioned beads. The depletion was verified by reduction in the ELISA binding of the depleted (flow-through) as compared to the undepleted plasma, with the corresponding wild type gp120 and thereby demonstrated the % of total gp120 specific antibodies depleted by the CD4bs probes. Briefly, RSC3, ΔN2mCHO, D368R were the CD4bs probes used for depletion. The plasma antibodies, before and after depletion, were tested for binding to the respective wild-type gp120 (HXB2 gp120 for both RSC3 and D368R depleted plasma and JRFL gp120 for ΔN2mCHO depleted plasma) (Fig 3). A 50–60% depletion of the CD4bs plasma antibodies was observed in both samples with D368R, confirming the presence of CD4bs antibodies in the two plasma samples. AIIMS 660 showed 52% depletion of ΔN2mCHO directed antibodies and 34% depletion of RSC3 directed antibodies of the total wild-type gp120. Plasma sample AIIMS 619 showed 23% depletion of ΔN2mCHO directed antibodies and 51% depletion of RSC3 directed antibodies of the total wild type gp120. Adsorption with the BSA coated beads was performed as specificity control (Fig 3) and depleted and undepleted plasma samples were checked for binding with HXB2 gp120. BSA-depleted plasma antibodies exhibited similar binding and neutralization as that of the corresponding undepleted plasma sample, confirming that there was no non-specific binding of plasma antibodies to BSA coated beads.

### CD4bs-dependent neutralization by plasma antibodies

The depleted plasma samples AIIMS 619 and AIIMS 660 were checked for CD4bs-directed neutralization against a panel of 7 pseudoviruses, 4 Subtype B (BaL.01,SF162, SC422661.8, JRFL) and 3 subtype C (MW965, DU156.12, DU422.1). Table 4 depicts the plasma CD4bs antibody ID50 titres after depletion on magnetic beads coated with various proteins: HXB2gp120, HXB2gp120-D368R, RSC3 and ΔN2mCHO. BSA coated beads were used as control. The proportion of antigen specific neutralization against a particular virus was calculated as percent reduction in ID50 as described previously [13]. Briefly, gp120 directed percentage neutralization against a particular virus was calculated using the formula: 100× [(ID50 titre of flow through of the BSA coated beads- ID50 titres of flow through of gp120 coated beads)/ (ID50 titres of flow through of BSA coated beads)]. Similarly, the D368R gp120, RSC3, and ΔN2mCHO directed neutralization was also calculated by substituting the ID50 values of flow through of respective antigen in the place of gp120 in the given formula. The CD4bs-directed % neutralization in the total gp120-directed neutralization was calculated as: [(percentage of HXB2 gp120 directed neutralization- percentage of HXB2 gp120-D368R directed neutralization)/ percentage of HXB2gp120 directed neutralization].

**Table 4:** ID50 values and percentage neutralization activity of plasma antibodies depleted using gp120 wild type and CD4bs-selective probes tested against different viruses.

		ID50 titres^a^						% Neutralization^b^				
Plasma ID	Pseudoviruses	Untreated Plasma	BSA	HXB2 gp120	HXB2 gp120 D368R	RSC3	ΔN2mCHO	HXB2 gp120 directed	HXB2 gp120 D368R directed	gp120 directed neutralization by CD4bs antibodies	RSC3 directed	ΔN2mCHO directed
**AIIMS 660**	**BaL.01**	1045	895	102	559	438	325	88%	37%	***57%***	***51%***	***63%***
	**SF162**	824	508	69	238	310	275	86%	53%	***38%***	***39%***	***45%***
	**JRFL**	521	516	158	273	388	319	69%	47%	***31%***	24%	***38%***
	**SC422661.8**	148	203	60	75	159	167	70%	63%	10%	21%	17%
	**MW965**	5425	6384	538	1296	5489	4967	91%	79%	13%	14%	22%
	**DU156.12**	375	407	105	129	301	296	74%	68%	8%	***26%***	***27%***
	**DU422.1**	586	602	160	187	569	485	73%	69%	5%	7%	19%
**AIIMS 619**												
	**BaL.01**	556	416	78	113	398	350	81%	72%	11%	4%	15%
	**SF162**	738	696	110	112	550	606	84%	83%	0%	20%	13%
	**JRFL**	330	258	69	78	210	245	73%	69%	0%	18%	5%
	**SC422661.8**	478	506	60	105	456	401	88%	79%	10%	9%	20%
	**MW965**	2890	2574	109	126	2257	2285	95%	95%	0%	12%	11%
	**DU156.12**	3367	2104	113	199	1812	1907	94%	90%	0%	13%	9%
	**DU422.2**	230	319	102	90	296	281	68%	71%	0%	7%	11%

Plasma samples were adsorbed to BSA, HXB2gp120, HXB2gp120-D368R, RSC3, and ΔN2mCHO-coupled beads and then tested in TZM-bl neutralization assays. The final plasma dilution in the assay was 1:60. Reciprocal neutralization ID50 titres are shown. In cases where no neutralization was observed at 1:60, a value of 60 is noted.

^a^ID50 of untreated plasma, plasma adsorbed on BSA coupled beads; plasma absorbed on HXB2 gp120 coupled beads; plasma adsorbed on HXB2 gp120-D368R beads; plasma adsorbed on RSC3 coupled beads; plasma adsorbed on ΔN2mCHO coupled beads;

^b^ % reduction in ID50 by HXB2 gp120 adsorption relative to that of BSA coated beads; Percentage reduction in ID50 by HXB2 gp120-D368R relative to BSA-coupled beads; Percentage of gp120-directed neutralization contributed by CD4bs antibodies; Percentage reduction in ID50 by RSC3 adsorption relative to BSA coated beads; Percentage reduction in ID50 by ΔN2mCHO adsorption relative to BSA coated beads.

Values in bold italics are considered significant; i.e., cases where gp120 adsorbs >50% of the neutralizing activity and >25% of the activity is directed to the CD4bs. The calculations are shown in the Materials and Methods section.

**Fig 4:**
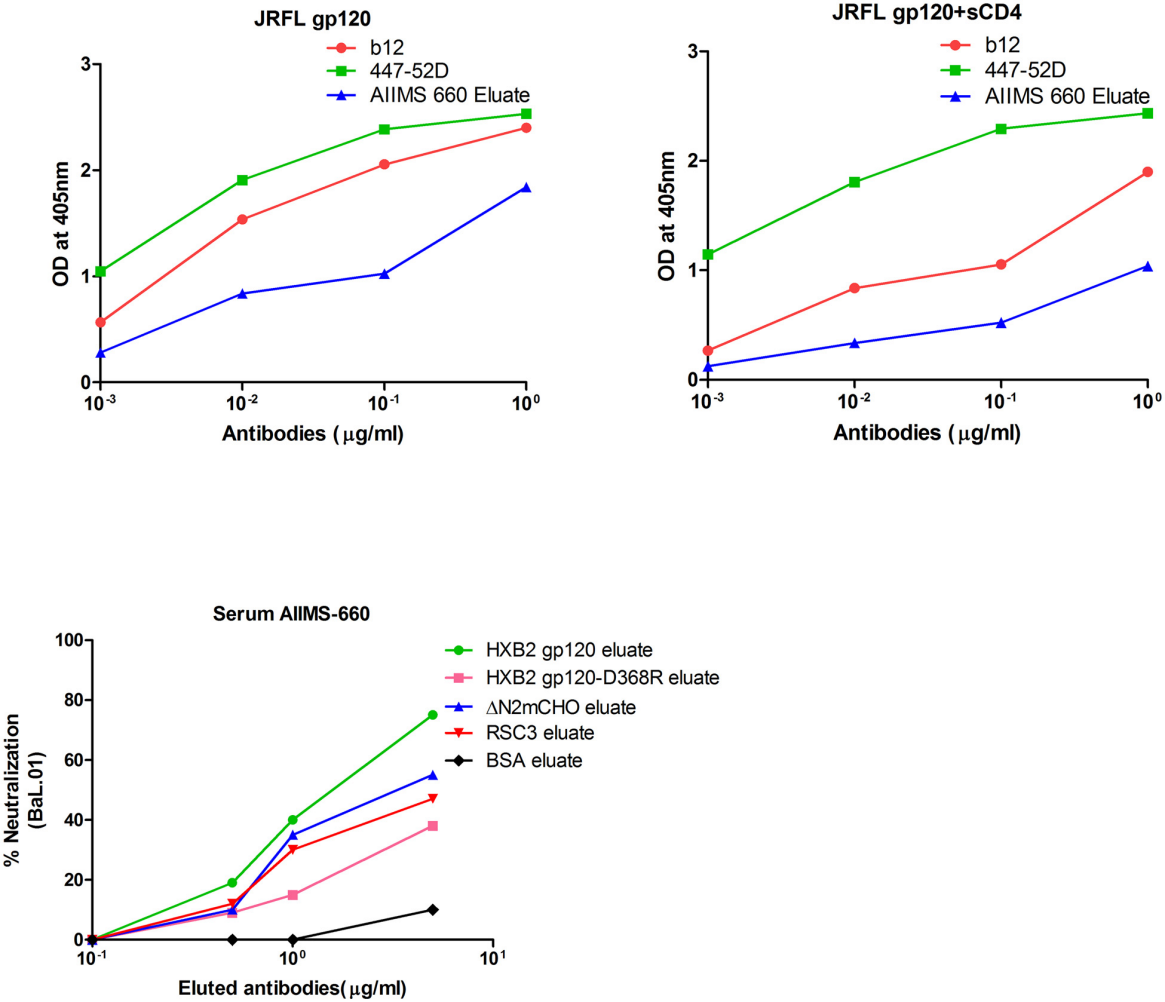
Competition ELISA binding and neutralization activity of eluted plasma antibodies from CD4bs probes coated on magnetic beads. The AIIMS 660 plasma antibodies were adsorbed onto magnetic beads coated with ΔN2mCHO, RSC3, HXB2 gp120, HXB2 gp120- D368R, BSA. Antibodies were eluted from ΔN2mCHO beads and were tested for competition ELISA binding in the presence and absence of soluble CD4. Panel 4A: b12 and 447-52D were used as positive and negative control respectively. Reduction in the binding of b12 and AIIMS 660 eluate in the presence of soluble CD4 shows CD4bs specificity. Panel 4B: Neutralization of HXB2 gp120 eluate and HXB2 gp120- D368R eluate, ΔN2mCHO and RSC3 eluate of AIIMS 660 with BaL.01 virus. The graph depicts percentage neutralization of BaL.01 virus with respect to plasma eluted antibody concentration of AIIMS 660.

HXB2 gp120 depleted flow through of both the CNP samples (AIIMS 619 and AIIMS 660) demonstrated considerable decrease in the neutralization capacity of the plasma antibodies, suggesting that most of the neutralizing activity is gp120 directed (ranging from 70% to 95% for different viruses). For plasma samples where >50% of the total neutralizing activity was reduced after adsorption with HXB2 gp120, a readout of >25% of CD4bs directed neutralizing activity was considered significant. Based on the ID50 reduction values, the percentage of CD4bs directed neutralization was calculated against the tested viruses (Table 4). Of the 7 viruses tested, 3 subtype B viruses (BaL.01, SF162 and JRFL) and 1 subtype C virus (DU156.12) exhibited varied neutralization susceptibility to AIIMS 660 plasma CD4bs directed antibodies, detailed as follows. BaL.01 was most susceptible, with 63%, 51% and 57% being ΔN2mCHO, RSC3 and CD4bs-gp120 directed neutralization respectively. For SF162 virus, 45%, 39% and 38% was ΔN2mCHO, RSC3 and CD4bs-gp120 directed neutralization respectively, while for JRFL, 38% and 31% was ΔN2mCHO and CD4bs-gp120 directed neutralization respectively. In the case of DU156.12, 27% and 26% was ΔN2mCHO and RSC3 directed neutralization respectively, by the CD4bs plasma antibodies of AIIMS 660. These results were further confirmed by testing the neutralization activity of AIIMS 660 eluates after adsorption onto the three CD4bs probes coated on magnetic beads. Briefly, the antibodies were eluted from the adsorbed HXB2 gp120 and HXB2 gp120-D368R, RSC3 and ΔN2mCHO coupled beads respectively and the concentration of the eluted antibody fraction was determined. Neutralization activity of the eluted antibodies was then tested against BaL.01 virus (Fig 4B). Around two fold reduction in neutralization activity by the HXB2 gp120-D368R eluate as compared to that of the HXB2 gp120 eluate confirmed the CD4bs neutralization dependence. Further, 55% and 47% CD4bs dependent neutralization was observed with the ΔN2mCHO and RSC3 eluates.

The depleted plasma sample AIIMS 619 showed gp120 directed neutralization, however CD4bs dependent neutralization was not observed against the subtype B and subtype C viruses tested.

### Competitive ELISA demonstrates CD4bs-directed antibodies in AIIMS 660 plasma

To further confirm the presence of CD4bs antibodies in the AIIMS 660 plasma, the antibodies adsorbed on the ΔN2mCHO coupled beads were eluted and tested for binding to the CD4bs of JRFL gp120 by competition ELISA with soluble CD4. The presence of sCD4 had no effect on the binding of 447-52D, as expected, sCD4 did inhibit the binding of b12 (used as a control) and also the ΔN2mCHO eluate of AIIMS 660 plasma to the JRFL gp120, confirming that antibodies present in the plasma are directed to the CD4bs region (Fig 4A).

## Discussion

This study attempted, for the first time, a systematic evaluation of the CD4bs specificities of the cross neutralizing plasma of Indian HIV-1 infected individuals and their contribution to viral neutralization. Consistent with previous findings, less than 10% of the plasma samples (11 of 140) exhibited substantial cross neutralization activity, with subtype C viruses (majority of the neutralized viruses belonged to tier 2) more efficiently neutralized than subtype B viruses [44]. Of these 11 samples, 5 contained CD4bs antibodies (as evidenced by reactivity to the CD4bs selective probes RSC3 and lack of reactivity to its CD4bs-defective variant RSC3Δ371I; significantly reduced binding with HXB2 gp120-D368R as compared to its wild type HXB2 gp120). Only one of these plasma showed binding to the ΔN2mCHO probe.

The antigenic diversity between subtype B and subtype C viruses could be a factor contributing to the differences in the neutralization specificities of the infected individuals of different populations as has been observed in earlier studies [10, 50, 51]. As postulated by Binley et al. [10], the CD4bs NAb activity in subtype C plasmas may plausibly be unaffected by the D368R mutation, unlike subtype B plasmas. Use of distinct well characterized CD4bs probes to map the plasma CD4bs antibody specificities will maximally increase the probability of picking up antibodies even with minor differences in their epitope specificities. We therefore used three well-characterised recombinant probes to identify the CD4bs specific antibodies in the seropositive plasmas exhibiting HIV-1 neutralizing activity. The specificity of the probes was confirmed by testing their binding reactivity with known bNAbs. Further stringency was maintained by positive scoring of the plasma antibodies for CD4bs site reactivity if they exhibited binding specificities with at least two of the three tested probes. Overall, we observed a varied distribution of CD4bs antibodies in the plasmas tested and that the CD4bs antibodies contributing to viral neutralization were rare in this study population.

Adsorption of the plasma antibodies of AIIMS 619 and AIIMS 660 using monomeric HXB2 gp120 and testing the neutralizing activity of the depleted plasmas revealed that gp120 (HXB2) directed NAbs majorly(>50%) contribute to neutralization of the subtype B and subtype C viruses tested. Our results suggest that monomeric gp120 directed plasma antibodies contribute to viral neutralization in these individuals and is similar to the findings of Gray *et al* [11]. However, earlier immunization studies, based on monomeric gp120 vaccines, have failed to elicit correlates of protection in the vaccinees [52, 53]. Some of the plausible determining factors could be their inability to mimic the trimeric envelope glycoprotein of virus in natural infection and the rapid clearance of antigen from the circulation contrary to that seen in chronically infected individuals, wherein antigenic stimulus persists for a prolonged period of time.

The presence of cross NAbs to the CD4bs in AIIMS 660 plasma confirms the CD4bs dependent neutralization activity of this plasma. One of the possible reasons for observing CD4bs neutralization dependence predominantly against subtype B viruses may be the use of CD4bs probes derived from subtype B viruses that are probably less effective in capturing antibodies that recognize subtype C specific determinants [10, 11]. The CD4bs dependent neutralization activity observed in the AIIMS 660 plasma identifies this individual as a potential candidate for the isolation of CD4bs NAbs.

An interesting observation of this study was that all the plasma samples (11/140; 8 from the present study and 3 from earlier cohort [44]) that exhibited cross-neutralizing activity were effective in neutralizing Du156.12 (subtype C). The extensive plasma cross neutralizing activity observed in the present and earlier studies [44, 54] in subtype C infected individuals suggests that subtype C epitopes are conserved in infecting viruses in a given population. This is further substantiated by the relatively limited genetic diversity observed in subtype C viruses from India [54]. Furthermore, mapping the CD4bs epitopes in subtype C viruses could provide useful information for identification of subtype C specific epitopes that may be incorporated into a polyvalent vaccine.

CD4bs is a conserved region in the viral envelope and known to be highly immunogenic. Longitudinal studies have revealed that VRC01-like CD4bs bNAbs develop later during the course of natural infection [42]. However, an intriguing finding in this study is that the plasma antibodies of AIIMS 619, a long term non-progressor, were effective in cross neutralization and showed binding reactivity with the CD4bs probes, but did not show CD4bs directed neutralization. This could be either due to the low titre of CD4bs antibodies or that the antibodies were not affinity matured and therefore did not contribute substantially to viral neutralization. Another possibility could be that non-CD4bs directed plasma antibodies may be the neutralization determinants in this plasma, which has not been addressed here and is a limitation of this study. Further, studies have shown that not all CD4bs antibodies necessarily have broad neutralizing activity [7].

Altogether our study adds to the information regarding the neutralization specificities present in Indian HIV-1 infected individuals exhibiting plasma cross neutralizing activity. This is the first time where we have reported the CD4bs dependent neutralization in an Indian HIV-1 infected donor. Majority of the potent CD4bs neutralizing antibodies have been so far identified from non-subtype C HIV-1 infected donors. Mapping of the neutralizing plasma for CD4bs antibody specificities in individuals infected with different viral strains will help in the design of CD4bs directed immunogens.

## Supporting Information

S1 Fig Coupling of recombinant proteins to magnetic beads. Integrity of the recombinant proteins coated onto the magnetic beads was confirmed by the binding of bNAbs using flow cytometry VRC01 was used for RSC3, b12 for HXB2 gp120 and ΔN2mCHO and 447-52D for HXB2 gp120-D368R. BSA coupled beads were used as a negative control for all the experiments. Data analysis was performed with Flow Jo software. Grey histograms represent BSA coated beads whereas red histograms indicate the binding of antibodies to the beads which indirectly shows coupling of the respective proteins onto the beads, thus confirming the integrity of coupled proteins.
(TIF)

S1 Table Non-neutralizing plasmas tested against pseudoviruses. Details of non-neutralizing plasmas of HIV-1 infected ART-naive individuals from India tested against a panel of subtype B and C viruses. The AIIMS ID of the plasma samples is provided at the top of the table. Plasma neutralization is shown as the reciprocal value of the ID50, which is the plasma dilution at which virus infectivity is inhibited to 50%. None of the above three plasma sample reached the ID50 titres.
(PDF)
