# Supplementary figures and images for: Identification of CD4-Binding Site Dependent Plasma Neutralizing Antibodies in an HIV-1 Infected Indian Individual
Source: PLoS One. 2015 May 11;10(5):e0125575. doi: 10.1371/journal.pone.0125575 (PMC4427266; doi:10.1371/journal.pone.0125575)

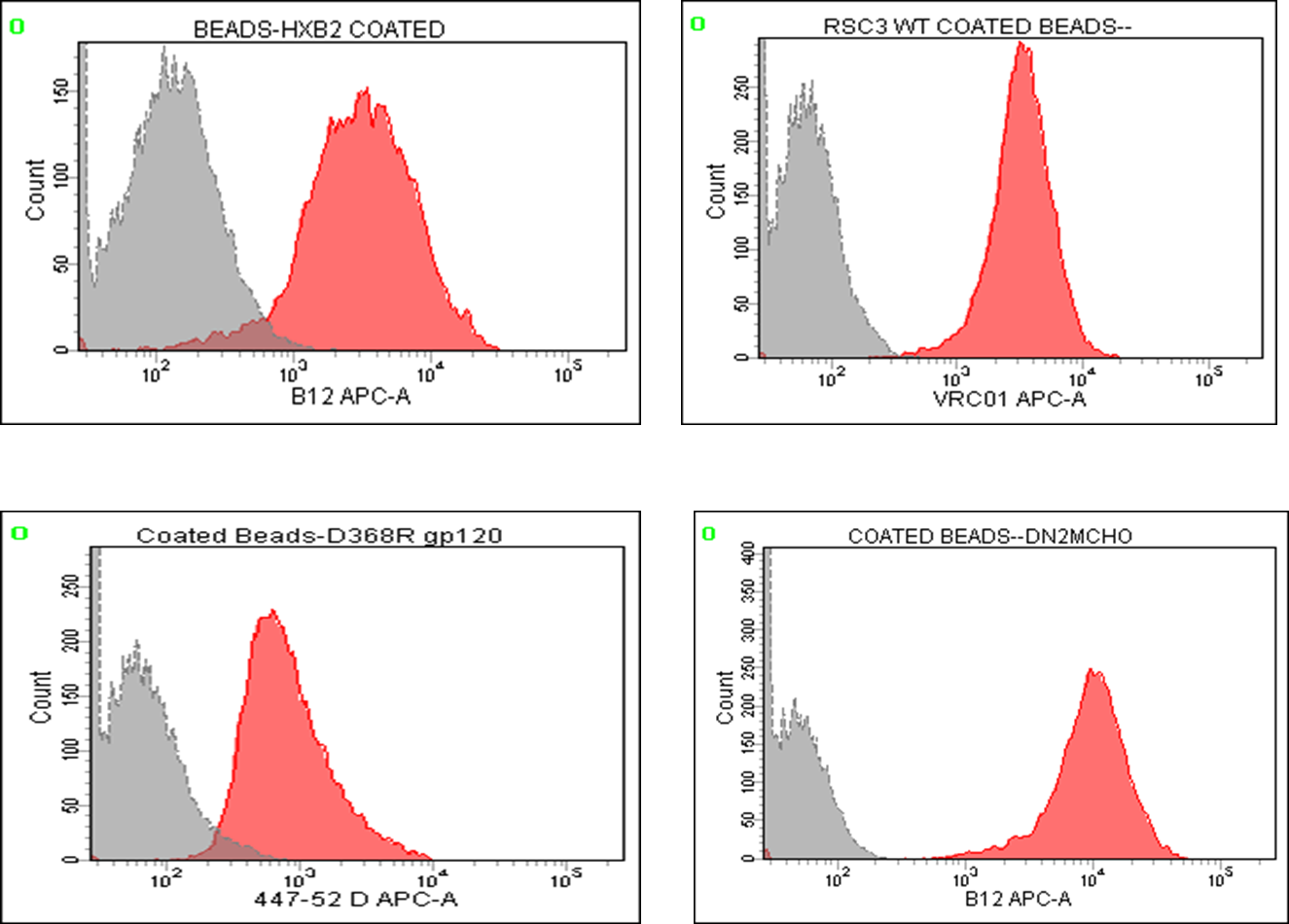

Supplement: S1 Fig — Integrity of the recombinant proteins coated onto the magnetic beads was confirmed by the binding of bNAbs using flow cytometry VRC01 was used for RSC3, b12 for HXB2 gp120 and ΔN2mCHO and 447-52D for HXB2 gp120-D368R. BSA coupled beads were used as a negative control for all the experiments. Data analysis was performed with Flow Jo software. Grey histograms represent BSA coated beads whereas red histograms indicate the binding of antibodies to the beads which indirectly shows coupling of the respective proteins onto the beads, thus confirming the integrity of coupled proteins. (TIF) [file pone.0125575.s001.tif]
