# Supplementary material for: Identification of CD4-Binding Site Dependent Plasma Neutralizing Antibodies in an HIV-1 Infected Indian Individual
Source: PLoS One. 2015 May 11;10(5):e0125575. doi: 10.1371/journal.pone.0125575 (PMC4427266; doi:10.1371/journal.pone.0125575)
Supplement: S1 Table — Details of non-neutralizing plasmas of HIV-1 infected ART-naive individuals from India tested against a panel of subtype B and C viruses. The AIIMS ID of the plasma samples is provided at the top of the table. Plasma neutralization is shown as the reciprocal value of the ID50, which is the plasma dilution at which virus infectivity is inhibited to 50%. None of the above three plasma sample reached the ID50 titres. (PDF) [file pone.0125575.s002.pdf]

S1Table: Non-neutralizing plasmas tested against pseudoviruses

Pseudovirus	Subtype	Tier	AIIMS-637	AIIMS-639	AIIMS-643
**Q168ENVa2**	A	2	<60	<60	<60
**BaL.01**	B	1	<60	<60	<60
**SF162**	B	1	<60	<60	<60
**RHPA4259.7**	B	2	<60	<60	<60
**TRO.11**	B	2	<60	<60	<60
**SC422661.8**	B	2	<60	<60	<60
**JRFL**	B	2	<60	<60	<60
**ZM109F.PB4**	C	1	<60	<60	<60
**25710**	C	1	<60	<60	<60
**MW965**	C	1	<60	<60	<60
**001428-2.42**	C	2	<60	<60	<60
**Du156.12**	C	2	<60	<60	<60
**Du422.1**	C	2	<60	<60	<60

S1Table: Details of non-neutralizing plasmas of HIV-1 infected ART-naïve individuals from India testedagainst a panel of subtype B and C viruses. The AIIMS ID of the plasma samples is provided at the top of the table. Plasma neutralization is shown as the reciprocal value of the ID50, which is the plasma dilution at which virus infectivity is inhibited to 50%. None of the above three plasma sample reached the ID50 titres
